# Anaphylaxis deaths in Ontario: a retrospective review of *cases* from 1986 to 2011

**DOI:** 10.1186/1710-1492-8-S1-A8

**Published:** 2012-11-02

**Authors:** Ya S Xu, Susan Waserman, Laura Harada, Monika Kastner

**Affiliations:** 1 Department of Pediatrics, McMaster University, Hamilton, Ontario, L8N 3Z5, Canada; 2 Department of Medicine, McMaster University, Hamilton, Ontario, L8N 3Z5, Canada; 3 Anaphylaxis Canada, Toronto, Ontario, M2J 5B4, Canada; 4 Li Ka Shing Knowledge Institute of St. Michael’s Hospital, Toronto, Ontario, M5B 1W8 Canada

## Background

Analysis of mortality data as a result of anaphylaxis may identify risk factors for severe reactions and highlight gaps in management. The primary objective of this study was to identify trends in and potential risk factors for fatal anaphylaxis in Ontario from 1986 to 2011.

## Materials and methods

We conducted a retrospective case study using two sources: 1) the Ontario Coroner's database from 2003-2011; and 2) unpublished death reports between 1986 and 2000 gathered by Anaphylaxis Canada. Outcomes of interest included type of allergen, nature of reaction and treatment. Analyses included descriptive statistics and frequency analysis (quantitative data) and grounded theory methodology (qualitative data).

## Results

There were 82 anaphylaxis deaths in Ontario in the last 25 years (63 deaths from 1986-2000; 19 deaths from 2003-2011). There was a decline in fatalities due to food allergy, from 32 deaths from 1986-2000 to 2 deaths during 2003-2011. Presumed deaths due to nuts also decreased within these time periods (17 vs 1 death). Among the 82 fatalities, an epinephrine auto-injector was prescribed for 17 patients (21%), only 9 of which (53%) carried it at the time of the reaction. Prior to hospital, only 19 patients (23%) received epinephrine (including by EMS).

## Conclusions

This retrospective case study of Ontario fatality indicates a decline in deaths due to anaphylaxis, and possibly decline in the number of deaths caused by food allergy, especially to nuts. The low proportion of patients who were administered epinephrine may indicate that more education is needed for both patients and EMS personnel regarding administration of epinephrine/auto-injectors.

